# Comparing the Output of an Artificial Intelligence Algorithm in Detecting Radiological Signs of Pulmonary Tuberculosis in Digital Chest X-Rays and Their Smartphone-Captured Photos of X-Ray Films: Retrospective Study

**DOI:** 10.2196/55641

**Published:** 2024-08-21

**Authors:** Smriti Ridhi, Dennis Robert, Pitamber Soren, Manish Kumar, Saniya Pawar, Bhargava Reddy

**Affiliations:** 1 Department of International Health, Johns Hopkins Bloomberg School of Public Health Baltimore, MD United States; 2 Qure.ai Bangalore India; 3 Innovators in Health Bihar India; 4 Qure.ai Mumbai India

**Keywords:** artificial intelligence, AI, deep learning, early detection, tuberculosis, TB, computer-aided detection, diagnostic accuracy, chest x-ray, mobile phone

## Abstract

**Background:**

Artificial intelligence (AI) based computer-aided detection devices are recommended for screening and triaging of pulmonary tuberculosis (TB) using digital chest x-ray (CXR) images (soft copies). Most AI algorithms are trained using input data from digital CXR Digital Imaging and Communications in Medicine (DICOM) files. There can be scenarios when only digital CXR films (hard copies) are available for interpretation. A smartphone-captured photo of the digital CXR film may be used for AI to process in such a scenario. There is a gap in the literature investigating if there is a significant difference in the performance of AI algorithms when digital CXR DICOM files are used as input for AI to process as opposed to photos of the digital CXR films being used as input.

**Objective:**

The primary objective was to compare the agreement of AI in detecting radiological signs of TB when using DICOM files (denoted as CXR_d_) as input versus when using smartphone-captured photos of digital CXR films (denoted as CXR_p_) with human readers.

**Methods:**

Pairs of CXR_d_ and CXR_p_ images were obtained retrospectively from patients screened for TB. AI results were obtained using both the CXR_d_ and CXR_p_ files. The majority consensus on the presence or absence of TB in CXR pairs was obtained from a panel of 3 independent radiologists. The positive and negative percent agreement of AI in detecting radiological signs of TB in CXR_d_ and CXR_p_ were estimated by comparing with the majority consensus. The distribution of AI probability scores was also compared.

**Results:**

A total of 1278 CXR pairs were analyzed. The positive percent agreement of AI was found to be 92.22% (95% CI 89.94-94.12) and 90.75% (95% CI 88.32-92.82), respectively, for CXR_d_ and CXR_p_ images (*P*=.09). The negative percent agreement of AI was 82.08% (95% CI 78.76-85.07) and 79.23% (95% CI 75.75-82.42), respectively, for CXR_d_ and CXR_p_ images (*P*=.06). The median of the AI probability score was 0.72 (IQR 0.11-0.97) in CXR_d_ and 0.72 (IQR 0.14-0.96) in CXR_p_ images (*P*=.75).

**Conclusions:**

We did not observe any statistically significant differences in the output of AI in digital CXRs and photos of digital CXR films.

## Introduction

An estimated 10.6 million people (133 per 100,000 population) were diagnosed with tuberculosis (TB) in the year 2022 which is an increase from the 10.3 million new cases reported in 2021 [1]. The number of deaths caused by TB in 2022 is estimated to be about 1.3 million [1]. Chest x-ray (CXR or chest radiographs) is a crucial tool in the TB diagnostic pathway, but the lack of qualified radiologists or health care professionals in interpreting CXRs and limited infrastructure for CXR facilities is a challenge in resource-limited settings which are not uncommon in high TB burden areas [2].

In light of increasingly promising evidence of the usefulness of computer-aided detection (CAD) technologies, such as those based on artificial intelligence (AI), the World Health Organization (WHO) has recommended their use as an alternative to human interpretation of digital CXR for screening and triage for pulmonary TB in individuals aged 15 years or older [3]. Many CAD tools intended for TB screening and triage using CXR use AI algorithms in the backend, and multiple such commercially available software devices are available for routine clinical use [4]. One of the commercially available AI CAD devices is qXR (version 3.2; Qure.ai). Many of these AI algorithms, including the algorithm in qXR, were trained primarily on digital CXR images using their Digital Imaging and Communications in Medicine (DICOM) files (soft copies) as inputs.

The diagnostic accuracy of qXR and many other similar commercially available devices has been evaluated previously in multiple studies [4-15]. A study conducted in a high TB-burden setting in Bangladesh reported that qXR has an AUC (area under the receiver operating characteristics curve) of 90.81% while also fulfilling the WHO’s Target Product Profile criteria of minimum 70% specificity at 90% sensitivity [4,16]. Another retrospective study conducted using CXRs from patients from Nepal and Cameroon reported that AI was better than human readers in detecting bacteriologically confirmed TB [9]. An independent evaluation of 12 different AI algorithms for TB detection in adults conducted in Vietnam found an AUC of about 82% for qXR [13]. In an active-case finding program conducted in India using both radiologists and qXR for CXR screening, a 15% increase in TB yield was found to be attributable to qXR [2]. WHO’s recommendation for the use of CAD in the screening and triaging of TB was primarily based on independent evaluations of multiple commercially available technologies, and qXR was among them [3].

While these studies provide substantial evidence supporting the use of AI in digital CXR images for TB screening and triage, there is limited evidence on the performance of such AI algorithms when photos of digital CXR films (hard copies) are taken using regular smartphones or when conventional plain film radiograph photos are used as inputs. This is important in resource-limited areas where there is a lack of digital CXR infrastructure [17-20]. Some studies have reported the use of CXR films and formats other than DICOM as inputs for training the TB detection AI algorithm, but it is not clear how exactly the films were fed as inputs to AI algorithms [21-23]. A recently published study of qXR reported negligible differences in performance in DICOM CXR files and photos of DICOM CXR films, but a formal statistical comparison was not performed [24].

In this retrospective cross-sectional analysis, we investigated if there is a significant difference in the agreement of qXR in detecting radiological signs of TB from CXR images in digital x-ray images (DICOM files) and their corresponding smartphone-captured CXR photos with majority consensus obtained from a panel of 3 radiologists.

## Methods

### AI CAD Device

qXR is an AI-based CXR interpretation software device [25]. The “TB detection” deep-learning algorithm in qXR is trained using roughly 100,000 digital CXR images (DICOM files) from individuals with microbiological confirmation for the presence or absence of tuberculous bacteria. qXR can be used to identify radiological signs of TB in frontal (posteroanterior or anterior-posterior views) CXR images of patients aged 6 years and older. A probability score between 0 and 1, denoting the likelihood of the presence of radiological signs of TB in a CXR image, is generated, and based on a set threshold, it classifies a CXR image for the presence or absence of radiological signs of TB. Ideally, the threshold is recommended to be calibrated by conducting on-site calibration studies prior to routine clinical use [26]. The manufacturer-recommended threshold is 0.5 and for this study, we used this threshold. Several other diagnostic accuracy studies of qXR have also used this threshold [11,14,15]. Typically, the input to qXR is a DICOM file of the CXR, but it can also process CXR images in JPEG or PNG formats. Throughout this paper, from here onward, we use the terms “AI,” “AI CAD device,” or “AI device” interchangeably, and all these terms denote qXR version 3.2.

### Study Design

This was a retrospective cross-sectional analysis. The following types of data were used for this analysis—deidentified and anonymous digital CXR images in their DICOM format (CXR_d_) and photos of digital CXR films captured using smartphones (CXR_p_), AI results in the form of numerical probability scores for both CXR_d_ and CXR_p_ images, and radiological majority consensus obtained from a panel of 3 radiologists. Except for the radiological majority consensus data, all other data were sourced retrospectively from historical records. The main objective was to evaluate and compare the agreement (quantified in the form of positive percent agreement [PPA] and negative percent agreement [NPA]) of the AI (qXR version 3.2) in detecting radiological signs of TB in CXR_d_ and CXR_p_ with majority consensus obtained from a panel of 3 radiologists. A 5% difference in PPA or NPA was considered to be a conservative clinically significant difference. We determined that a sample of 1146 CXR pairs will provide about 80% power to detect a difference in PPA or NPA of 5%, assuming a conservative PPA (or NPA) of 80% and 75%, respectively, in CXR_d_ and CXR_p_ images for paired observations data with a moderate correlation of 0.5 [27]. We included 1300 CXR pairs initially and after applying exclusion criteria, the final analysis included 1278 CXR pairs, meaning that our analysis had more than 80% power to detect a minimum difference of 5%.

The inclusion criteria were CXR_d_ and CXR_p_ pairs of frontal CXR images from patients aged 6 years or older and the availability of majority consensus from the radiologist panel. Microbiological reference standards for TB confirmation were not available in the retrospective data. We excluded any duplicate CXR images from the same patients.

### Details of the Retrospective Data

The CXRs used for this analysis were originally captured in their digital form as part of a different TB screening project (Stop TB REACH Wave 7 grant initiative) in Bihar, India, conducted during the period from July 2020 to January 2021 [28]. During this project, community health workers performed doorstep screenings using a structured questionnaire in the regional language (Hindi) to identify individuals exhibiting any symptoms indicative of TB. Those with symptoms were referred to nearby health centers for further evaluation. All symptomatic individuals were advised to undergo CXR examinations as part of the routine TB diagnostic pathway. The CXRs used in this analysis were done at 4 private x-ray centers during the Stop TB REACH Wave 7 project. A grantee of this project proposal, Innovators in Health, a nonprofit organization, was involved in this data collection.

**Figure 1:**
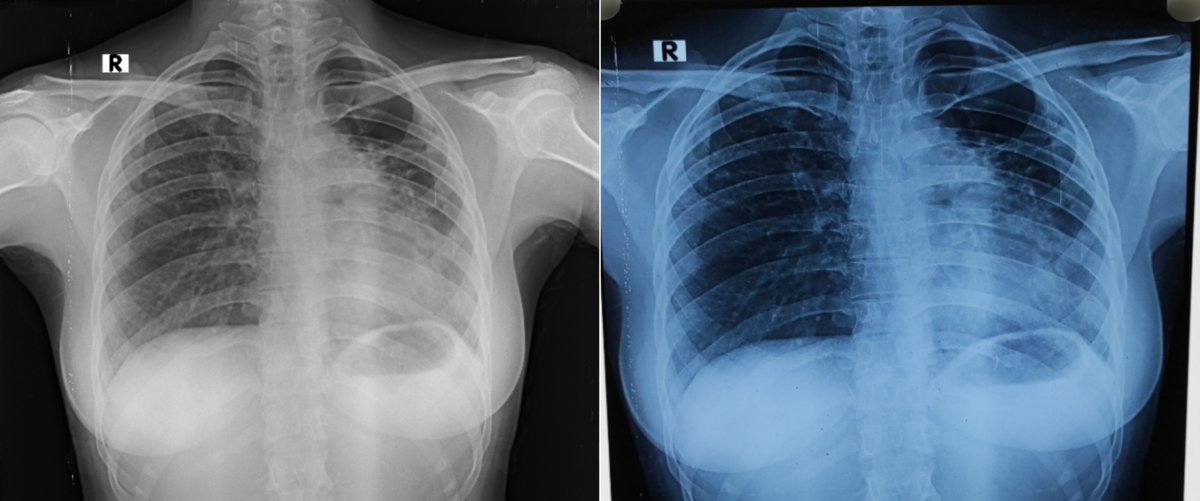
A digital CXR image (on the left) viewed in a DICOM viewer and its corresponding smartphone-captured photo (on the right). CXR: chest x-ray; DICOM: Digital Imaging and Communications in Medicine.

Innovators in Health staff captured photos of the digital CXR films (CXR_p_) using regular smartphones (Xiaomi Redmi Note 5 Pro and Samsung Note 7 Pro). There were specific instructions as to how to capture photos of the CXR films. These included placing the film on a lightbox in a dark room, switching off the flashlights of the phones, phones to be kept in parallel to the film, apex and base of the lungs visible in the camera, and the captured photo not being rotated or flipped. Illustrative guidance on how to capture photos is available in Multimedia Appendix 1. An example of a CXR_d_ image and its corresponding CXR_p_ image is shown in Figure 1.

Thus, for each digital CXR file in DICOM format (CXR_d_), we had a corresponding photo of the digital CXR film captured using a smartphone (CXR_p_) in JPEG format. During the TB screening project, AI was also used for TB screening, and thus, historical records also contained the AI results. Before any statistical analysis, any personally identifiable information was removed. The retrospective data included both images of each CXR pair, the AI probability score (a numeric value between 0 and 1) indicating the likelihood for the presence of radiological signs of TB for each CXR pair, the age of the patient at the time of CXR acquisition, and gender.

### Establishment of Majority Consensus by Human Readers

The majority consensus establishment was done as part of this study. This was performed separately after the collection of the retrospective data. A panel of 3 general radiologists with 3 to 10 years of postresidency experience in interpreting CXRs formed the radiologist panel. These radiologists had significant experience in interpreting CXR images for TB diagnostic workups in the high TB burden setting of India. They were not part of the TB screening project from which the retrospective data were collected. All radiologists were blinded to the “CXR_d_-CXR_p_ pair” information, AI result, and clinical history. The order of the CXR_d_ and CXR_p_ images were randomized for each reader. The majority opinion on the presence or absence of radiological signs of TB in the CXR was considered the majority consensus. Of the 3 radiologists, 2 of them initially independently read all CXR pairs. They classified each CXR into one of the following 2 categories: the presence of radiological signs of TB and the absence of radiological signs of TB. Thus, for each CXR pair, we obtained 2 readings from 1 radiologist, one each for CXR_d_ and CXR_p_ images. If all 4 readings for a CXR pair from the 2 radiologists were the same (concordant CXR pairs), this was considered the majority consensus. The third radiologist read discordant CXR pairs. Thus, for the discordant CXR pairs, we had 6 readings in total from 3 radiologists (3 each for CXR_d_ and CXR_p_). For analysis purposes, we used 3 different majority consensus (Table 1): majority consensus on CXR_d_ (MC_d_), majority consensus on CXR_p_ (MC_p_), and a global majority consensus (MC_g_).

**Table 1:** Types of majority consensus and their descriptions.

Majority consensus type	Definition
Majority consensus on CXR_d_ (MC_d_)	Majority vote by the radiologist panel for the digital CXR^a^ images (CXR_d_).
Majority consensus on CXR_p_ (MC_p_)	Majority vote by the radiologist panel for the photos of digital CXR films (CXR_p_).
Global majority consensus (MC_g_)	Majority vote by the radiologist panel for all pairs of CXR images. If there was a tie (eg, 3 TB^b^ positive votes and 3 TB negative votes), the majority vote for the digital CXR_d_ was considered as the final consensus decision.

^a^CXR: chest x-ray.

^b^TB: tuberculosis.

### Statistical Analysis

Since the original CXR source was digital, MC_d_ was used for the primary objective of comparing the PPA and NPA of AI in CXR_d_ versus CXR_p_ images. Calculations of PPA and NPA are like that of sensitivity and specificity respectively, but the terminologies of PPA and NPA indicate that the majority consensus of human readers used in this study is a nonreference standard [29]. Secondary analyses based on MC_p_ and MC_g_ are also reported. The manufacturer-defined threshold of 0.5 was applied to the probability scores obtained from the AI CAD device to obtain a categorical decision for the presence or absence of radiological signs of TB in each CXR. Any indeterminate test results or missing values from AI, if occurred, were reported. The point estimates of PPA and NPA are reported along with the exact binomial 95% CI. McNemar’s test was used to compare the PPA and NPA of AI between CXR_d_ and CXR_p_ images [30]. Agresti-Min 95% CI is reported for differences in PPA and NPA [31]. AUC was estimated based on empirical method, and DeLong’s 95% CI for AUC is reported [32]. It is to be noted the use of the term “AUC” may be misleading in that it uses a reference standard, but we want to emphasize that, unlike PPA and NPA, no such standard terminologies were available to report a metric, like AUC, when a nonreference standard is used. DeLong’s test was used to compare AUCs [32]. We also compared the distribution of AI probability scores in CXR_d_ and CXR_p_. We also report agreement statistics between results from CXR_p_ and CXR_d_ images by the radiologists and AI CAD device by providing point estimates and 95% CI for Cohen κ, prevalence and bias-adjusted κ, Gwet’s AC1 [33], and overall percentage agreement [34]. McNemar’s chi-square test for the symmetry of rows and columns in a 2D contingency table was also performed to investigate the difference in AI output (using the default threshold of 0.5) in CXR_d_ and CXR_p_ images. Sensitivity analysis by changing the threshold to 0.3, 0.4, 0.6, and 0.7 are also reported. All the statistical analysis was done using R (version 4.2.1; R Core Team).

### Ethical Considerations

The study was approved by an independent ethics committee of the Royal Pune Independent Ethics Committee (IEC RPIEC041023). Informed consent requirement was not required due to the retrospective nature of the study. Only deidentified data were used for any analysis. Participants were not compensated as this was a retrospective study using only deidentified CXR images.

## Results

### Overview

**Figure 2:**
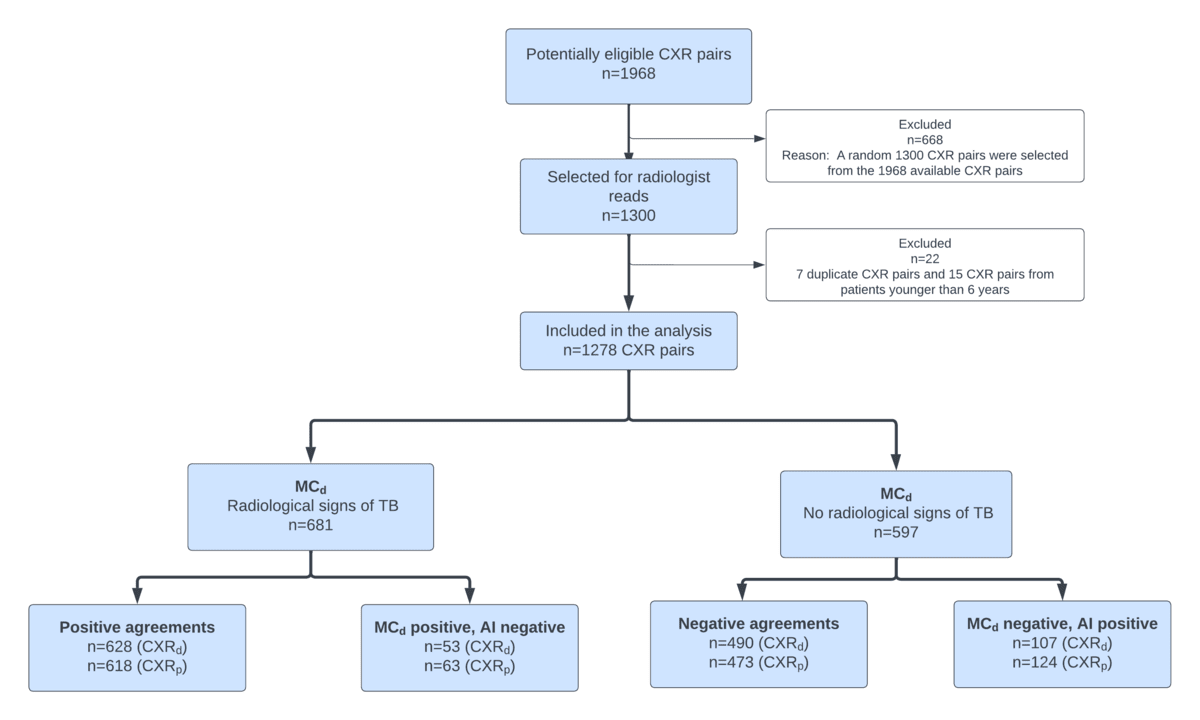
Data flow diagram. AI: artificial intelligence; CXR: chest x-ray; CXR_p_: photo of the digital CXR film; MC_d_: majority consensus of the radiologists based on digital CXR images (CXR_d_); TB: tuberculosis.

A total of 1300 CXR pairs were considered for the analysis. After applying inclusion and exclusion criteria, 22 (7 duplicates and 15 from patients younger than 6 years) CXR pairs were excluded. Thus, a total of 1278 CXR pairs from 1278 distinct patients were included in the final analysis (Figure 2). There were no indeterminate results or missing values from the AI.

### Baseline Characteristics and Summary Statistics of Majority Consensus

The CXR_p_ images in JPEG format were generated by photographing the CXR_d_ films using the Xiaomi Redmi Note 5 Pro and Samsung Note 7 Pro. The CXR_d_ images were originally acquired using x-ray machines manufactured by Fujifilm. Both CXR_d_ images (in DICOM format) and CXR_p_ images (in JPEG format) had a minimum 1440×1440 pixels resolution. The size of the CXR_p_ files ranged from 1.2 to 4.8 MB.

The mean age of the patients from whom the CXRs were sourced was 44.2 (SD 17.3; median 46, IQR 29-61) years. A total of 1232 (96.4%) CXR pairs were from patients older than 15 years. Gender information was not available in the metadata of 12 CXRs, and in the remaining 1266 CXRs, 659 (52%) CXRs were males. Among the 1278 CXR pairs, 812 (63.5%) CXRs had a complete agreement (both CXR_d_ and CXR_p_ interpretations were the same) between the 2 radiologists. The other 466 (36.5%) CXR pairs were additionally sent for reading by a third radiologist. Based on MC_d_, 681 (53.3%) CXRs were positive, and the rest, 597 (46.7%) CXRs, were negative for the presence of radiological signs of TB.

### Agreement With Majority Consensus

**Figure 3:**
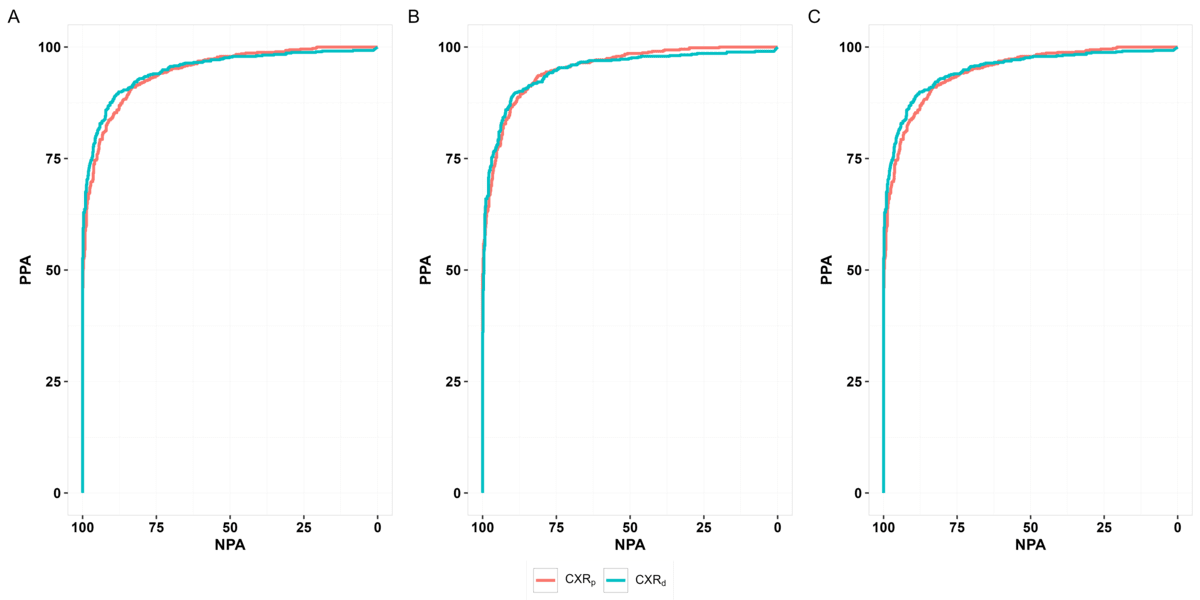
(A) AUC curves using MC_d_, (B) AUC curves using MC_p_, and (C) AUC curves using MC_g_. AUC: area under the receiver operating characteristics curve; CXR: chest x-ray; MC_d_: majority consensus on CXR_d_; MC_g_: global majority consensus; MC_p_: majority consensus on CXR_p_; NPA: negative percent agreement; PPA: positive percent agreement.

**Figure 4:**
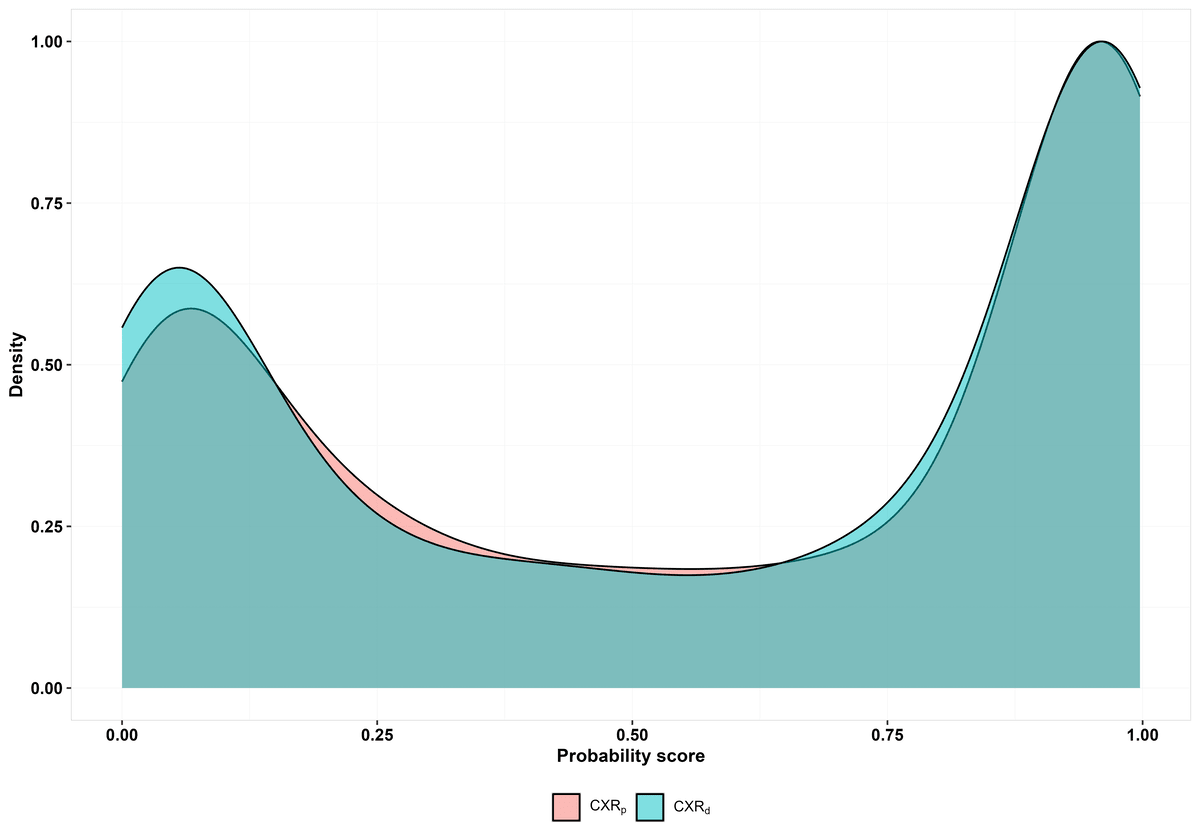
Distribution of probability scores from artificial intelligence. CXR: chest x-ray.

Using MC_d_, the PPA of AI was found to be 92.22% (95% CI 89.94-94.12) and 90.75% (95% CI 88.32-92.82), respectively, for CXR_d_ and CXR_p_ images (difference: 1.47; *P*=.09). The NPA of AI was 82.08% (95% CI 78.76-85.07) and 79.23% (95% CI 75.75-82.42), respectively, for CXR_d_ and CXR_p_ images (difference: 2.85; *P*=.06) using MC_d_. Both PPA and NPA differences were statistically insignificant in all comparisons using MC_d_, MC_p_, and MC_g_ (Table 2). Using MC_d_, the AUC of AI in CXR_d_ and CXR_p_ images were found to be 95.09% (95% CI 93.95-96.24) and 93.67% (95% CI 92.39-94.95), respectively, and this difference was statistically significant (difference: 1.42; *P*=.01). AUC curves are shown in Figure 3. The differences in AUC were all statistically insignificant while using MC_p_ and MC_g_ (Table 2). The mean absolute difference in the probability scores from the AI in CXR_d_ and CXR_p_ was 0.09 (SD 0.15; median 0.03, IQR 0.01-0.11). The distribution of the probability scores is shown in Figure 4. The median of the AI probability score was 0.72 (IQR 0.11-0.97) in CXR_d_ and 0.72 (IQR 0.14-0.96) in CXR_p_ images (*P*=.75).

**Table 2:** Positive and negative percent agreements and AUC^a^ of AI^b^.

			MC_d_^c^				MC_p_^c^			MC_g_^c^		
			CXR_d_^d^		CXR_p_		CXR_d_		CXR_p_	CXR_d_		CXR_p_
**PPA^e^**												
	P_a_/P^f^, n/n	628/681		618/681		589/626		592/626		618/666	614/666	
	PPA%^g^ (95% CI)	92.22% (89.94 to 94.12)		90.75% (88.32 to 92.82)		94.09% (91.94 to 95.80)		94.57% (92.49 to 96.21)		92.79% (90.40 to 94.52)	92.19% (89.89 to 94.11)	
	Δ%^h^ (95% CI)	1.47% (–0.27 to 3.21)		—^i^		–0.48% (–2.13 to 1.17)		—		0.60% (–1.13 to 2.33)	—	
	*P *value	.09		—		.56		—		.49	—	
**NPA^j^**												
	N_a_/N^k^, n/n	490/597		473/597		506/652		502/652		495/612	485/612	
	NPA%^g^ (95% CI)	82.08% (78.76 to 85.07)		79.23% (75.75 to 82.42)		77.61% (74.21 to 80.75)		76.99% (73.57 to 80.17)		80.88 (77.54 to 83.92)	79.08% (75.64 to 82.24)	
	Δ% (95% CI)	2.85% (–0.18 to 5.87)		—		0.62% (–2.31 to 3.53)		—		1.80% (–1.19 to 4.79)	—	
	*P *value	.06		—		.68		—		.24	—	
**AUC**												
	AUC%^g^ (95% CI)	95.09% (93.95 to 96.24)		93.67% (92.39 to 94.95)		94.80% (93.54 to 96.05)		95.19% (94.13 to 96.25)		94.99% (93.81 to 96.16)	94.59% (93.46 to 95.73)	
	Δ% (95% CI)	1.42% (0.33 to 2.51)		—		–0.39% (–0.67 to 1.45)		—		0.40% (–1.40 to 0.61)	—	
	*P *value	.01		—		.47		—		.44	—	

^a^AUC: area under the receiver operating characteristic curve.

^b^AI: artificial intelligence.

^c^MC_d_, MC_p_, and MC_g_: Majority consensus on CXR_d_, majority consensus on CXR_p_ and global majority consensus, respectively of human readers. PPA, NPA and AUC comparisons using all three majority consensus types are reported in the table.

^d^CXR: chest x-ray.

^e^PPA: positive percent agreement.

^f^P_a_/P: the number of positive agreements (P_a_) and the total number of positive CXRs (P) as per majority consensus of radiologists.

^g^PPA%, NPA%, and AUC%: point estimates of PPA, NPA, and AUC, respectively.

^h^Δ%: point estimates of percentage point differences in PPA%, NPA%, and AUC% between CXR_d_ and CXR_p_ images.

^i^Not applicable.

^j^NPA: negative percent agreement.

^k^N_a_/N: the number of negative agreements (N_a_) and the total number of negative CXRs as per majority consensus of radiologists (N).

### Agreement Statistics of AI and Radiologists in Interpreting CXR_d_ and CXR_p_ Images

McNemar’s chi-square test for symmetry of rows and columns in the 2D contingency table (Table 3) of AI decisions (presence or absence of radiological signs of TB based on the default threshold of 0.5) in CXR_d_ and CXR_p_ images returned a statistically insignificant (*P*=.58) result. A sensitivity analysis was performed by changing the threshold to 0.3, 0.4, 0.6, and 0.7, and all of these returned statistically insignificant results (McNemar’s chi-square test *P*=.40, .40, .99, and .80, respectively) suggesting no significant differences in the binary decisions outputted by the AI in CXR_d_ and CXR_p_ images.

The agreement (Cohen κ) between the CXR_d_ and CXR_d_ results of the AI was 0.81 (95% CI 0.77-0.84), and for the 2 radiologists who read all the pairs of CXRs images were 0.53 (95% CI 0.46-0.56) and 0.85 (95% CI 0.82-0.88), respectively. In the subgroup of 466 discordant CXR pairs, the agreement was 0.67 (95% CI 0.60-0.74) and 0.62 (95% CI 0.55-0.70) for the AI and the radiologist, respectively (Table 4). Overall, the AI had the same results in 90.53% (95% CI 88.79-92.08) of the CXR pairs. There was strong agreement (Cohen κ=0.84; 95% CI 0.82-0.87) in the majority consensus on CXR_d_ (MC_d_) and CXR_p_ (MC_p_) images. Radiologist 2 (Table 4) had a strong agreement (Cohen κ=0.85; 95% CI 0.82-0.88). Gwet’s AC1 and prevalence and bias-adjusted κ also showed similar trends to that of Cohen κ estimates.

**Table 3:** Contingency table of AI^a^ results in CXR_d_^b^ and CXR_p_ images.

	AI CXR_p_ positive result	AI CXR_p_ negative result
AI CXR_d_ positive result	678	57
AI CXR_d_ negative result	64	479

^a^AI: artificial intelligence.

^b^CXR: chest x-ray.

**Table 4:** Agreement statistics of AI^a^ and radiologists in interpreting digital CXRs^b^ and photos of corresponding digital CXR films.

		Cohen κ (95% CI)	Gwet’s AC1 (95% CI)	PABAK^c^ (95% CI)	Percentage agreement (95% CI)
**All CXR pairs (n=1278)**					
	AI	0.81 (0.77-0.84)	0.81 (0.78-0.85)	0.81 (0.78-0.84)	90.53% (88.79-92.08)
	Radiologist 1^d^	0.53 (0.46-0.56)	0.55 (0.51-0.60)	0.53 (0.48-0.57)	76.37% (73.94-78.67)
	Radiologist 2^d^	0.85 (0.82-0.88)	0.85 (0.82-0.88)	0.85 (0.82-0.88)	92.64% (91.07-94.02)
	MC_d_^e^ with MC_P_	0.84 (0.82-0.87)	0.84 (0.82-0.87)	0.84 (0.81-0.87)	92.2% (90.65-93.66)
**Discordant CXR pairs (n=466)**					
	AI	0.67 (0.60-0.74)	0.67 (0.60-0.74)	0.67 (0.60-0.73)	83.48% (79.79-86.73)
	Radiologist 3^f^	0.62 (0.55-0.70)	0.68 (0.61-0.74)	0.65 (0.58-0.72)	82.62% (78.87-85.95)

^a^AI: artificial intelligence.

^b^CXR: chest x-ray.

^c^PABAK: prevalence and bias-adjusted κ.

^d^Radiologists 1 and 2 are the two radiologists who read all pairs of CXR images.

^e^MC: majority consensus.

^f^Radiologist 3 read only the discordant CXR pairs.

## Discussion

### Principal Results

We observed no statistically significant differences in the PPA and NPA of AI in digital CXR images (input to AI here is a DICOM file of the digital CXR) and their corresponding photos of digital CXR films (input to AI here is a smartphone-captured photo of the digital CXR film in JPEG format). Since the study was adequately powered, and the differences in PPA and NPA fell outside of the critical region, this can be considered as a sign that the output of AI does not differ between digital CXRs (CXR_d_) and photos of digital CXR films (CXR_p_) based on the Neyman-Pearson approach of hypothesis testing [35]. The AUC of AI was significantly higher in digital CXR images (95.09 vs 93.67; *P*=.01). This percentage point difference of 1.42% in AUC is likely small and may not be clinically significant, and this trend of significantly different AUC was not observed in the secondary analyses using MC_p_ and MC_g_. Moreover, the variance of AUC is comparatively much lesser than that of a proportion like PPA or NPA [36] and it is very likely that our data had more than 80% power to detect a minimum detectable difference in AUC lesser than 5% (overpowered). This is probably the reason why we observed a statistically significant difference in AUC even for such a small effect size of 1.42%. Figure 4 illustrates that the distribution of AI probability scores is quite similar in CXR_d_ and CXR_p_ images. We observed a high proportion (n= 681, 53.3%) of patients whose CXR_d_ were identified with radiological signs of TB as per majority consensus, and this could be due to the fact that these patients had already undergone a symptomatic assessment of TB, and only those who were identified with symptoms suggestive of TB had undergone the digital CXR investigation as part of the TB screening project from which the data used for analysis were retrospectively extracted.

A strong intrarater agreement (Cohen κ=0.81) in the results of CXR_d_ and CXR_p_ images was observed for AI; one radiologist had a weak agreement (κ=0.53), the other radiologist had a strong agreement (κ=0.85). This is indicative of the known inter- and intrareader variabilities of human readers [24-27]. Although the agreement in the majority consensus was strong (κ=0.84), the observed reader variabilities could be the reason why we observed no substantially improved agreement between the majority consensus of CXR_d_ (MC_d_) and CXR_p_ (MC_p_) as compared to a senior radiologist (Radiologist 2). The differences in PPA and NPA of AI in CXR_d_ and CXR_d_ images were tested using interpretation results from Radiologist 2, as a sensitivity analysis, and all the differences were statistically insignificant. AI was also not completely immune to intrareader variability, as indicated by Cohen κ of 0.81 and percentage agreement of 90.53%. However, this demonstrated a strong agreement by AI in interpreting CXR_d_ and CXR_p_ images. Agreement statistics of AI were comparable to that of Radiologist 2 who was more experienced than Radiologist 1. In the subgroup analysis using only the discordant CXR pairs, both AI (κ=0.67) and the radiologist (κ=0.62) had a moderate agreement. The PPA and NPA of AI were found to be always >90% and >75%, respectively, in all comparisons for the overall sample. We also found that there are no statistically significant differences in the output of AI in CXR_d_ and CXR_p_ images at various thresholds of 0.3, 0.4, 0.5, 0.6, and 0.7. Our findings suggest that even a simple photo of a digital CXR film captured by following simple instructions may be sufficient for the AI. This is valuable in scenarios where digital files (soft copies) are not available to the patient or in environments where digital displays may not be practical due to limited technological infrastructure.

### Limitations

This study has several limitations. The first limitation is that the original source of the CXR_p_ was still digital CXR films and thus cannot be considered conventional CXR plain films per se. Hence, this study cannot be used to draw any inferences about the performance of AI in a conventional plain chest radiograph. We used smartphone-captured photos of the digital CXR films against a lightbox in order to enable a head-to-head comparison of the results from AI. New studies using conventional plain film radiographs are needed to evaluate the performance of AI in such settings. The second limitation is that we did not have a microbiological reference standard for TB. Instead, we used a radiological majority consensus using a panel of 3 radiologists for our analysis and interreader variabilities can impact estimations of PPA and NPA. We tried to mitigate this, at least partly, by using a panel of 3 radiologists instead of 1.

### Comparison With Prior Work

One study of the same AI CAD device reported no large differences in the performance of AI in digital and photographs of digital CXR films [24]. The study population of this work was different, and the statistical comparisons were descriptive and not inferential. The smartphones used to capture photos were also different. This work provides inferential evidence and reports additional comparisons with human readers while at the same time corroborating the finding that there is no difference in the performance of AI in digital CXR images and the photos of digital CXR films.

There are plenty of peer-reviewed publications reporting the diagnostic accuracy of AI algorithms for digital CXR-based TB detection and this has already been discussed in the introduction of this paper and a systematic review is available [8]. Some other studies have reported the use of “CXR films” in training their AI models. Nijiati et al [21] reported minimum sensitivity and specificity of about 93% for all 3 different AI models although this was performed only on an internal testing data set. Liu et al [22] reported an AUC of 0.76 + .006 in an external test set. Lakhani and Sundaram [23] trained an AI model using a CXR data set containing both PNG and DICOM images and reported a very high AUC of 0.99 in the internal test set. However, in these studies, it is not clear whether the authors took photos of the films or not to be fed as inputs to AI.

### Conclusions

We observed no statistically significant differences in the output of the AI CAD device in digital CXR images and corresponding smartphone-captured photos of digital CXR films. A simple-to-follow set of instructions can be used to capture photos of digital CXR films to ensure a stable performance of the AI CAD device if only digital CXR films are available.

## Appendix

Multimedia Appendix 1 Instructions used for capturing photos of chest x-ray films.

## Abbreviations

AI: artificial intelligence
AUC: area under the receiver operating characteristic curve
CAD: computer-aided detection
CXR: chest x-ray
DICOM: Digital Imaging and Communications in Medicine
MC: majority consensus
NPA: negative percent agreement
PPA: positive percent agreement
TB: tuberculosis
WHO: World Health Organization

## Data Availability

The data set used for statistical analysis during this study is available from the corresponding author upon reasonable request.
