# Supplementary material for: Comparing the Output of an Artificial Intelligence Algorithm in Detecting Radiological Signs of Pulmonary Tuberculosis in Digital Chest X-Rays and Their Smartphone-Captured Photos of X-Ray Films: Retrospective Study
Source: JMIR Form Res. 2024 Aug 21;8:e55641. doi: 10.2196/55641 (PMC11375380; doi:10.2196/55641)

Steps to follow

❶❷❸

❶ Before clicking the picture

- Make sure the light box is switched on

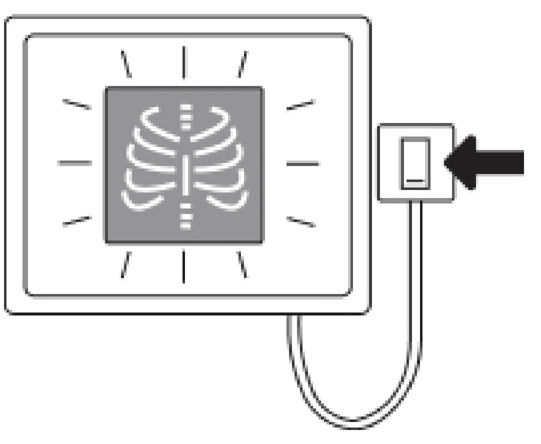

- Make sure the film is clipped to the light box

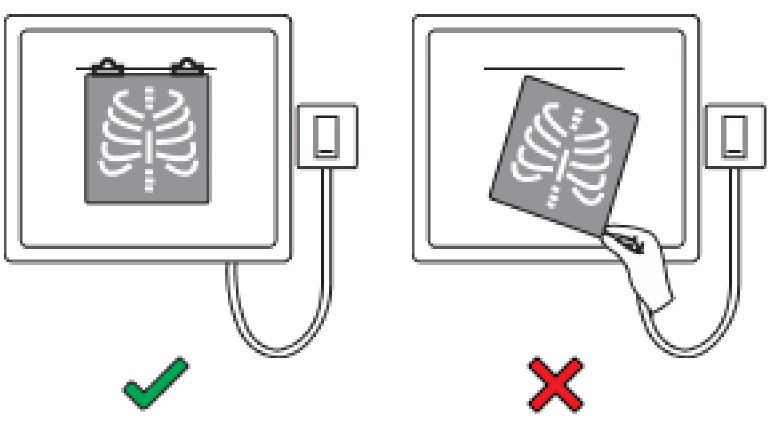

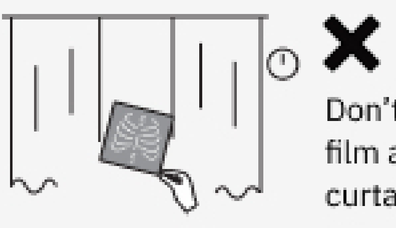

Don't click pictures of the film against wall, floor, curtain etc.

- Make sure the room is dark

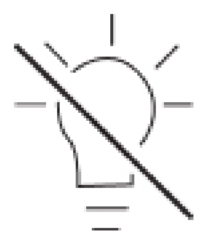

Turn off the lights

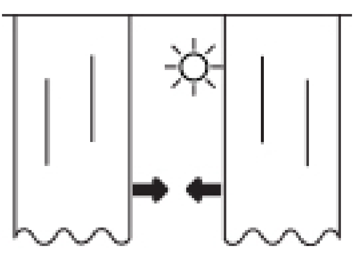

Close the curtains

- Stand within 2 ft of the light box

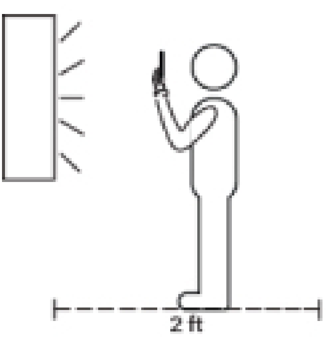

❷ While clicking the picture

- Keep your phone's flashlight off

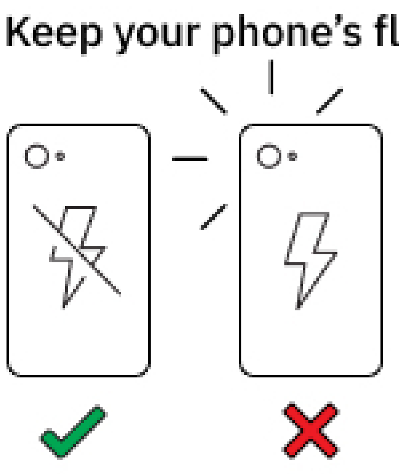

- Make sure your phone is parellel to the film

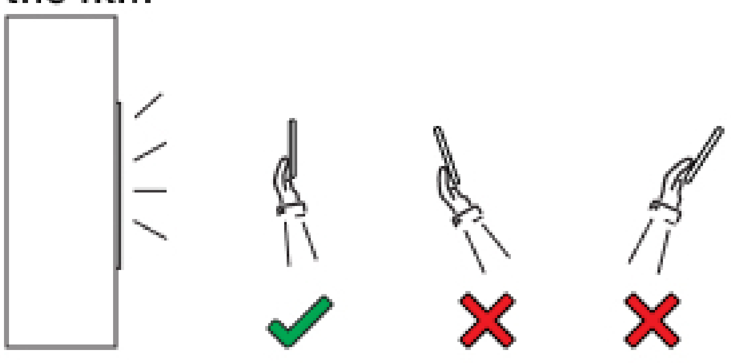

- Train the focus pointer onto the darker area of the image

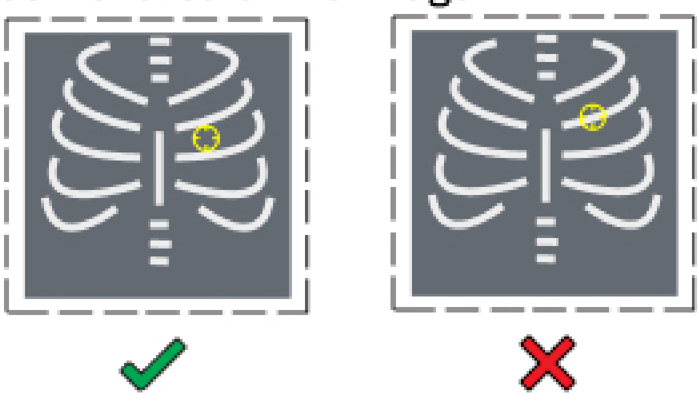

- Make sure the Apex & Base of the lungs are visible in the image

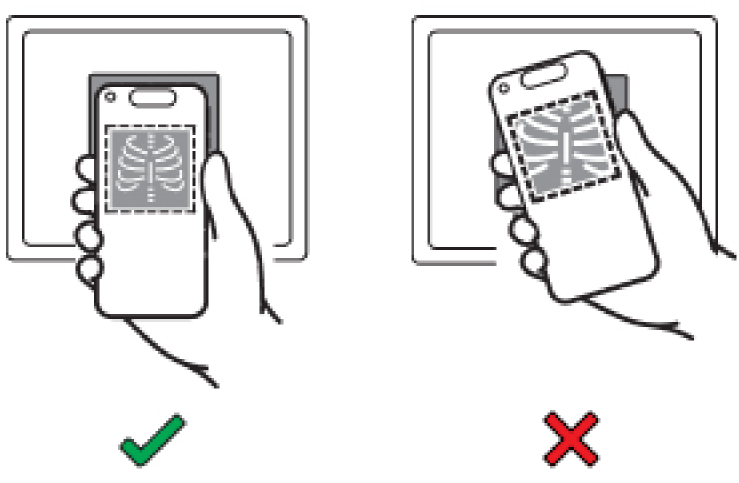

❸ After clicking the picture

- Crop the image such that..

  Only the lung area in the film should be in the frame

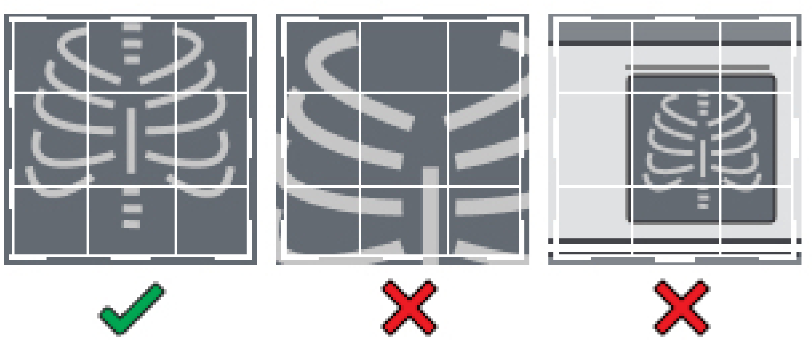

- Make sure the images you upload are not rotated or flipped

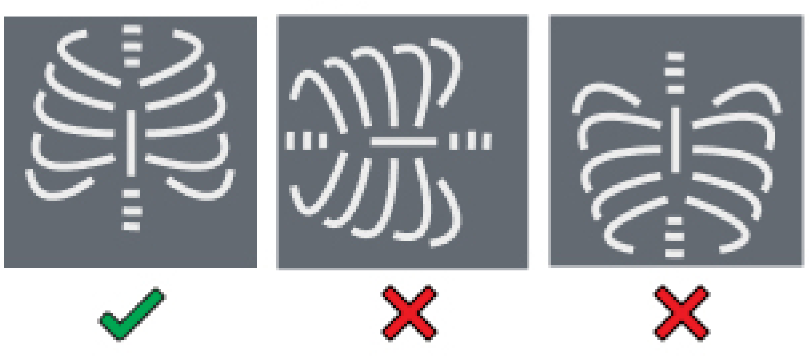

Supplement: Multimedia Appendix 1 [file formative_v8i1e55641_app1.pdf]
